# Middle Georgia Medical College

**Published:** 1860-07

**Authors:** 


THE MIDDLE GEORGIA MEDICAL COLLEGE has been inaugurated at Griffin, Georgia, making the fourth medical school in that State.

